# A randomised, double-blind, placebo-controlled trial to assess the postprandial dose-dependent effects of wild blueberries on metabolic and cognitive outcomes following a high-carbohydrate breakfast

**DOI:** 10.1007/s00394-026-03974-0

**Published:** 2026-05-26

**Authors:** Lucy R. Ellis, Dominic O’Connor, Haseena Khan, Louise Dye, Christine Boesch

**Affiliations:** 1 School of Psychology, University of Leeds, Leeds, UK; 2 School of Food Science and Nutrition, University of Leeds, Leeds, UK; 3 Carnegie School of Sport, Leeds Beckett University, Leeds, UK; 4 Division of Psychology and Mental Health, University of Manchester, Manchester, UK; 5 Institute of Sustainable Food, University of Sheffield, Sheffield, UK

**Keywords:** Blueberry, Glucose, Anthocyanins, Satiety, Blood pressure, Cognition

## Abstract

**Purpose:**

Despite equivocal human study data, anthocyanin-rich blueberries are associated with positive glycaemic effects which could benefit satiety and other cardiometabolic outcomes. The objective of this study was to examine the dose-dependent effects of freeze-dried wild blueberries on postprandial glucose response simultaneously with changes in satiety, blood pressure and cognitive function.

**Methods:**

Twenty-four healthy participants (F = 22), mean BMI 22.9 kg/m^2^ and mean age 28 years were recruited to a randomized crossover study in which they received a carbohydrate-rich breakfast with a wild blueberry or placebo-matched drink (250 mL) providing 0, 150, 300 or 450 mg anthocyanins. At baseline and at 30 min intervals during the 3-h postprandial phase, blood pressure, subjective appetite ratings and gastrointestinal satiety hormones from a plasma sample (up to 150 min) were recorded. Blood glucose was measured using continuous glucose monitoring. Cognitive performance was assessed at baseline and 90 min post-meal consumption.

**Results:**

Postprandial glucose and insulin levels declined in a dose-dependent manner. Medium and high doses showed significantly lower glucose and insulin compared to control during the first hour. Concurrently, satiety hormones GLP-1, PYY, and GIP demonstrated significant increases, most pronounced at the highest dose (450 mg anthocyanins), although subjective appetite was unaltered. Cognitive assessments using the Visual Verbal Learning Test, Corsi test and Rapid Visual Information Processing test revealed no significant intervention effects and blood pressure was unaffected.

**Conclusion:**

Wild blueberry effects on postprandial glucose and appetite hormone responses were evident at anthocyanin doses of 300 mg and above but did not impact other outcomes in healthy adults. Further studies should include individuals with metabolic and/or cognitive vulnerability in longer term interventions to confirm benefits of wild blueberries.

**Supplementary Information:**

The online version contains supplementary material available at 10.1007/s00394-026-03974-0.

## Introduction

Satiety is governed by a complex interplay of social, psychological, and physiological responses. Satiety is impacted by the postprandial response to a meal which is also considered to be a strong predictor of metabolic health [1]. Incretin hormones are key modulators of appetite regulation and glucose control which trigger reward regions in the brain in response to different nutrients. Notably, glucagon-like peptide-1 (GLP-1) and peptide YY (PYY) are secreted from enteroendocrine L-cells in the jejunal-ileal region and the distal colon in response to food ingestion [2]. In brief, GLP-1 amplifies insulin secretion from pancreatic β cells after food ingestion and PYY slows gastric emptying and gastrointestinal motility thereby increasing ileal absorption. Slower gastric emptying contributes to improved satiety and a more gradual glucose response following meals. Control of glucose is achieved through different mechanisms such as limiting absorption of glucose from the gut, increasing secretion of insulin and enhancing glucose uptake in cells and muscles [3]. By improving satiety response to a meal, this may reduce the risk of obesity and other metabolic conditions such as type 2 diabetes mellitus or metabolic syndrome.

Anthocyanins are a polyphenol subgroup responsible for the red, purple, and blue pigmentation in various fruits and vegetables [4]. They are found in dark fruits such as blueberries, cherries, and blackcurrants, which typically contain high concentrations—ranging from approximately 60–400 mg per 100 g fresh weight (FW) in blueberries, 30–80 mg/100 g FW in cherries, and 130–400 mg/100 g FW in blackcurrants. In comparison, common anthocyanin-containing vegetables such as aubergine (40–90 mg/100 g FW, primarily in the skin), red onions (20–50 mg/100 g FW), and red cabbage (100–300 mg/100 g FW) generally exhibit lower concentrations.

In vitro and animal studies demonstrate significant impact of anthocyanins on postprandial glycaemia and modulation of gut hormones. A 14-week supplementation of male mice with 40 mg/kg anthocyanins increased circulating GLP-1 by 98% [5]. The murine dose of 40 mg/kg corresponds to a human equivalent dose of ~ 224 mg/day, and this level of anthocyanin intake is achievable through dietary supplementation and may warrant further investigation in human trials. Notably, DPP-IV activity, an inhibitor of GLP-1, was decreased in GLUTag cells (a cellular model of GLP-1 secreting enteroendocrine L-cells) incubated with anthocyanins. The increases in GLP-1 may be anthocyanin-specific, as cyanidin, delphinidin and protocatechuic acid upregulated GLP-1 by 53%, 33% and 53%, respectively, in GLUTag cells [5]. Together, these gastrointestinal hormones contribute to body mass and obesity reduction by acting as anorexigenic signals through the gut-brain axis by promoting satiety and reducing overall appetite. However, most of the current evidence is derived from animal studies.

Meta-analyses suggest that repeated anthocyanin intake (≥ 320 mg/day for ≥ 8 weeks) may improve glycaemic markers—fasting blood glucose, HbA1c, and 2-h postprandial glucose in individuals with type 2 diabetes [6]. However, the diabetic participants in the included studies were taking some form of diabetic medication, which makes it difficult to determine if the effects were due to the medication, the anthocyanins, or their concomitant intake. No significant improvements were observed in healthy individuals, highlighting the potential for greater benefit in metabolically challenged populations.

Several studies have shown that anthocyanin-rich berries such as blackcurrants, cranberries, and blueberries can reduce postprandial glucose responses in human trials [7–10]. Blueberries in particular contain high concentrations of anthocyanins with some cultivars reaching 534 mg/100 g fresh weight [11].

Studies investigating chronic blueberry intake have reported mixed findings, with metabolic benefits appearing to depend on the population studied. For instance, consumption of a blueberry smoothie containing 668 mg of anthocyanins twice daily for six weeks improved insulin sensitivity in insulin-resistant individuals, although fasting glucose levels remained unchanged [12]. In contrast, daily consumption of 160 g of fresh blueberries (~ 220 mg polyphenols) or an equivalent amount of blueberry powder (288 mg polyphenols) for one week did not significantly affect blood pressure in healthy young adults [13]. However, in healthy older adults, supplementation with freeze-dried blueberry providing 302 mg of anthocyanins for twelve weeks improved endothelial function, as assessed by flow-mediated dilation, and was also associated with improvements in memory and executive function [14].

However, findings of single dose studies are not consistent. In one acute crossover study (n = 18), ingestion of 400 mg anthocyanins from blueberries did not significantly affect postprandial glucose, insulin, gastrointestinal hormones, or perceived appetite in healthy participants [15]. Similarly, intake of one cup of blueberries (364 mg anthocyanins) significantly increased blood glucose levels at 60 min post-intake. After 2 h, both glucose and insulin were lower compared to an isocaloric placebo, when consumed alongside a high-carbohydrate, high-fat meal. However, this did not translate into effects on blood pressure in older adults with metabolic syndrome [16].

A recent postprandial study using a wild blueberry extract (222 mg anthocyanins) reported acute reductions in systolic and diastolic blood pressure as well as faster executive function reaction times in healthy adults [17]. However, no acute cognitive effects (attention, memory, executive function) were observed in a recent trial using a blueberry drink, although the presence of microbial anthocyanin metabolites in plasma was associated with better cognitive performance [18]. Cognitive effects following blueberry interventions remain inconsistent and may be domain specific [19, 20].

There is a need for studies to define dose-dependent effects on glycaemia and satiety of polyphenol interventions, which has been highlighted in a recent review [21]. Therefore, the purpose of this study was to evaluate effects of increasing doses of anthocyanin-containing wild blueberry on postprandial glucose, appetite and gastrointestinal hormone response. A secondary aim was to determine the impact of blueberry intake on blood pressure and measures of cognitive function.

## Materials and methods

### Study design and ethics

This acute postprandial study conformed to a randomised, double-blind, crossover design in which participants consumed three different blueberry-rich test drinks or a placebo alongside a high carbohydrate bread-based meal. The study was approved by the University of Leeds, School of Psychology Research Ethics Committee (PSYC-855) and performed in accordance with the ethical standards as laid down in the 1964 Declaration of Helsinki and its later amendments. Research staff involved in the collection and analysis of the data remained blinded to the treatment randomisation until the study and all analyses were complete. An impartial member of staff within the Human Appetite Research Unit (HARU) prepared the intervention drinks. Participants were randomised electronically to condition order in a counterbalanced method with each test day separated by a minimum of 7 days.

### Participants

Hafiz et al., (2022) calculated that 18 participants were required to detect a change in postprandial glucose [22]. To account for potential dropouts, issues with blood withdrawal and balanced randomisation, a total of 24 healthy participants were recruited for the current study by advertisement within the University of Leeds. Healthy males and females aged between 18 and 56 years of normal/slightly elevated body weight (BMI < 27 kgm^2^) were eligible for this study. Participants were excluded if they did not have sufficient fluency of the English language due to non-native speakers using different cognitive processes during cognitive tests [23]. Additional exclusion criteria included metabolic comorbidities (high blood pressure (140/90), high cholesterol, and diabetes), clinical depression or any gastrointestinal disorders, known allergy/adverse reaction to blueberries, following a weight loss diet or having lost more than 6 kg in the last 4 months, smoking, medication use (other than contraceptives), peri-menopausal/menopausal women. Participants attended a screening visit to determine eligibility for the study. At screening, participants completed a general health and food questionnaire and completed practice versions of the cognitive test battery to minimise practice effects [24]. Data on height and weight were collected to calculate BMI. Participants were requested to maintain their normal diet but to eliminate consumption of typical anthocyanin-containing foods (berries, grapes, juices and wine) for 24 h prior to each test visit. On the evening prior to each test day, participants were provided with a standardized evening meal (Supplementary Table 1, 840 kcal, 90 g carbohydrates) to be consumed between 20:00 and 21:00 h in accordance with a previous study [25]. After this time, only consumption of water was permitted.

### Study day procedures

**Fig. 1:**
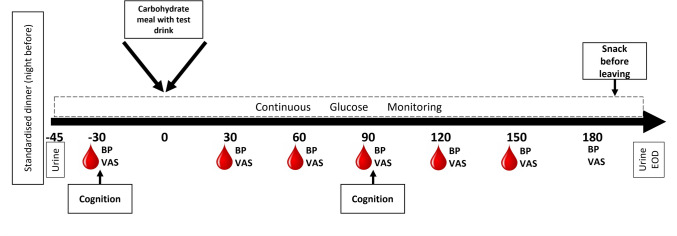
Timeline of procedure on test days. BP (blood pressure), VAS (visual analogue scale), EOD (End of Day Questionnaire)

On the morning of each test day, participants attended the Human Appetite Research Unit (HARU) at the University of Leeds, between 8:00 and 10:00 am following a 12 h fast and provided a urine sample in a sample cup (max of 200 mL urine), with boric acid added for stability. Participants were instructed to rest for 15 min before they filled in a visual analogue scale (VAS) using pen and paper relating to their appetite and mood and had their blood pressure taken. A cannula was inserted into the participant’s antecubital vein, typically in the right arm, by a trained phlebotomist and a 5 mL blood sample was taken. The first cognitive test battery (session 1) commenced after participants’ first blood sample. After completion of the cognitive test battery, participants were given the study drink and carbohydrate meal and asked to consume this within 15 min (Supplementary Table 1). Blood pressure and VAS were measured at every 30 min for 3 h, at 30, 60, 90, 120, 150 and 180 min post consumption of the study meal. Blood samples were drawn at the same time points up to 150 min. The second cognitive test battery (session 2) was completed at 90 min post consumption to coincide with peak anthocyanin concentration in circulation according to recent research [26]. Blood glucose was measured across the day using a continuous glucose monitor (CGM) which had been fitted to participants’ triceps 1–2 days before the first (and the third) study visit. Once the session was completed, the cannula was removed, another urine sample collected, and participants were offered complimentary snacks before leaving the lab. At the end of the day, participants were asked to complete an end of day questionnaire regarding their general health across the day and to assess any gastrointestinal problems. The study day procedures are outlined in Fig. 1.

### Intervention product

The intervention drinks were prepared as combinations of freeze-dried wild blueberry powder and placebo powder to provide 150, 300 and 450 mg anthocyanins (total of 30 g powder), named as low (LBB), medium (MBB), high (HBB) (Supplementary Table 2). The placebo was composed of maltodextrin and fructose to match sugar, carbohydrate and fibre content, with synthetic blueberry flavour added. Wild blueberry and placebo powders were provided by the Wild Blueberry Association of North America (WBANA). All beverages were prepared by an independent member of staff in the HARU in a standard shaker cup by thoroughly mixing the powder with 250 mL of Buxton mineral water and then transferred to an opaque drink container with a straw to give to the participants to maintain blinding. Together, the intervention drinks and breakfast (2 slices of Warburtons Toastie bread, toasted), provided ~ 80 g of total carbohydrates.

### Blood pressure and blood glucose

Blood pressure was measured at baseline and at 30-min intervals across the test sessions using an Omron M2 automatic blood pressure monitor. Measurements were taken with the participant in a seated position, in duplicate and then the value averaged. Interstitial glucose (ISG) was measured by a continuous glucose monitor (CGM, FreeStyle Libre Pro iQ, Abbott GmbH, Germany). The monitor remained in place for the duration of two visits, and then a new CGM was attached for the remaining visits. Although ISG values have been demonstrated to reliably reflect glucose concentration, there is a 5 to 10 min physiological delay in ISG glucose response to changes in blood glucose [27]. The CGM records glucose data every 15 min, with data downloaded from the device using Freestyle Libreview software (version 3.15) once the study visits were completed.

### Cognitive tests

Participants performed a 20-min cognitive test battery consisting of the Visual Verbal Learning Test immediate and delayed (VVLT, verbal memory), Corsi Block Tapping Test (Corsi, spatial memory), Rapid Visual Information Processing (RVIP, sustained attention). Outcome measures for the cognitive tests were as follows, VVLT (number of correct immediate and delayed recall, proactive and retroactive interference), Corsi (reaction time and accuracy of correct responses) and RVIP (accuracy and reaction time of correct responses and false alarms). The tests were developed and administered in the order presented below using E-Prime (version 2.0, Psychology Software Tools, Pittsburgh, PA). Parallel forms of each test were administered in a counterbalanced method to minimise practice effects and overlearning [24]. The cognitive tests for this study were chosen because they have shown sensitivity to other anthocyanin intervention studies [28].

### Subjective measures of appetite

Subjective ratings of appetite, fullness, and hunger were measured before consumption of the test beverages and at 30-min intervals across the study morning using a validated visual analogue scale (VAS) [29]. The scale used a 100 mm line ranging from 0 (“not at all”) to 100 (“extremely”). The participants were required to indicate how they felt on the following measures: hunger, fullness, contentedness, sleepiness, concentration, energy, and stress.

### Biological sampling and analyses

Blood samples were collected in EDTA vacutainer tubes with and without protease and centrifuged immediately for 10 min at 1500 rpm. The resulting plasma was stored in aliquots at − 80 °C for subsequent analyses. On the day of measurement, samples were carefully defrosted on ice, diluted accordingly, and the target analytes, insulin, GLP-1, PYY and GIP, quantified using enzyme linked immunosorbent assay (ELISA) according to the manufacturer’s instructions. Insulin was measured using RayBio® Human Insulin ELISA (Cambridge Bioscience, Cambridge, UK). The concentration of GLP-1 was determined using the GLP-1 multispecies ELISA kit (ThermoFisher Scientific, Invitrogen, Loughborough, UK) which measures the biologically active form of GLP-1 (7-36) amides). Finally, PYY and GIP were determined using ELISA kits from Sigma-Aldrich (Dorset, UK). Urine samples were analysed using fast-blue assay to determine total polyphenol concentration [30]. All laboratory analyses were performed prior to unblinding the intervention group assignments. All analytes were measured in duplicate against respective standard curves and presented as means with standard error.

### Statistical analysis

All data were first processed in Microsoft Excel (365) and then analysed using Statistical Package for Social Sciences (SPSS v29, Chicago, IL, USA). Participant characteristics were tabulated and descriptive statistics reported. All data was assessed for normality using box plots, the Shapiro–Wilk test and by visual inspection of residual plots. Linear mixed models (LMM), with drink (control, LBB, MBB or HBB), session/time and visit as fixed factors and participants as a random factor were performed. Baseline performance was included as a fixed factor with age and BMI added as covariates. For cognitive data, additional covariates of concentrations of gastrointestinal hormones, blood pressure and glucose were added as covariates as there is evidence that these factors can influence cognitive function [31, 32]. The model was performed using the Akaike’s Information Criterion (AIC) where a smaller AIC value indicates a better model fit [33]. Bonferroni adjusted pairwise comparisons explored any significant effects.

For blood glucose, incremental area under the curve (iAUC) was calculated using the trapezoidal method [34]. Data are expressed as means ± SE unless otherwise stated. Effects were considered significant if *p* < 0.05.

## Results

### Participant characteristics

**Fig. 2:**
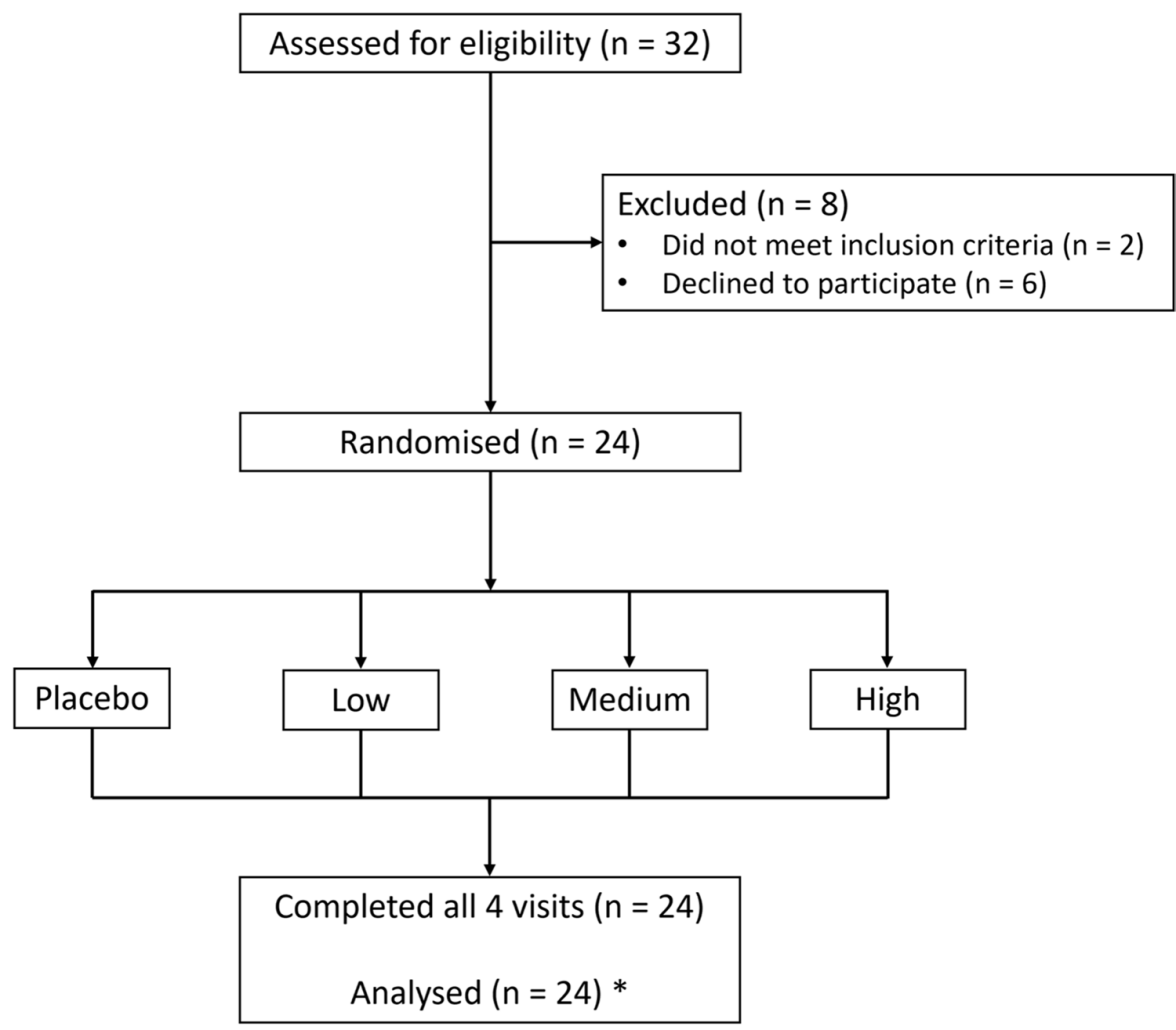
Flowchart of screening and randomisation of participants into the study. * 2 participants declined to provide blood samples but provided full data for all other outcomes

Of the 51 individuals who indicated their interest in study participation, 32 attended a screening visit. As detailed in the study flowchart (Fig. 2), two participants were excluded as they did not meet inclusion criteria (inability to perform cognitive tests to sufficient standard due to English language constraints) and 6 declined to participate. In total, 24 participants signed an informed consent form and completed all arms of the postprandial trial. Participants were predominantly female (n = 22) with a mean BMI of 22.9 kg/m^2^ (SE ± 2.1) and mean age of 28 years (21–38 years), detailed in Table 1. No adverse effects were reported across the study.

**Table 1:** Characteristics of participants at baseline (female n = 22, male n = 2)

Subject characteristics	Mean (standard error)
Age (years)	28.3 (1.2)
Weight (kg)	61.6 (2.1)
BMI (kg/m^2^)	22.9 (2.1)
Fasting glucose (mmol/L)	5.21 (0.17)
Baseline systolic blood pressure (mmHg)	104.6 (1.63)
Baseline diastolic blood pressure (mmHg)	70.9 (1.57)

### Blood glucose, insulin and blood pressure

**Fig. 3:**
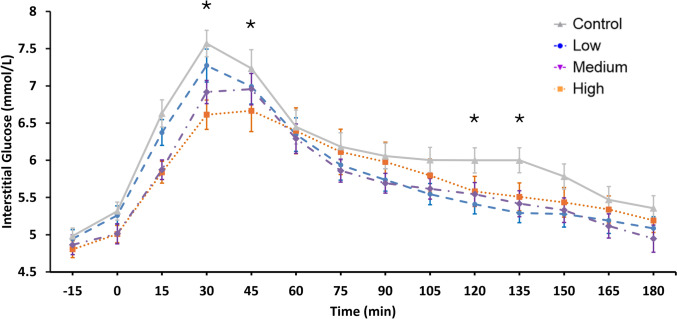
Postprandial glucose concentration after ingestion of the different blueberry interventions or control drink alongside a carbohydrate-rich meal. Significant differences (*) were observed between the HBB and control and the MBB and control at 30 and 45 min postprandial and at time points 120 and 135 min for glucose between the LBB and control and the MBB and control, controlling for baseline glucose in the analyses. A pairwise test with Bonferroni correction was performed for significant time points. Data are presented as mean ± SE

There were no significant main effects of condition, time, visit or interaction effects for blood pressure or heart rate. The postprandial changes in interstitial glucose after the test drinks and carbohydrate meal are shown in Fig. 3. A significant main effect of time was observed F(14, 890) = 51.427, *p* < 0.001. There was no main effect of visit F(3, 63) = 4.774, *p* = 0.315. The main effect of condition failed to reach significance F(3, 63) = 2.395, *p* = 0.077, however a condition x time interaction was observed F(39, 819) = 1.692, *p* = 0.006. Pairwise comparisons showed that there were significant differences in glucose concentrations across multiple time points, mainly in the initial 30–45 min of the postprandial period. Both MBB and the HBB significantly decreased glucose at 30 and 45 min relative to the control. In addition, the LBB and MBB interventions had significantly lower glucose at 120 and 135 min relative to the control. For AUC, a significant main effect of drink was observed at 60 min F(3, 73) = 3.915, *p* = 0.012. Pairwise comparisons revealed that there was a significant difference between the control and HBB (Supplementary Fig. 1). No other significant effects were observed for 90 min, 120 min or total AUC between drinks.

### Postprandial hormones and satiety

**Fig. 4:**
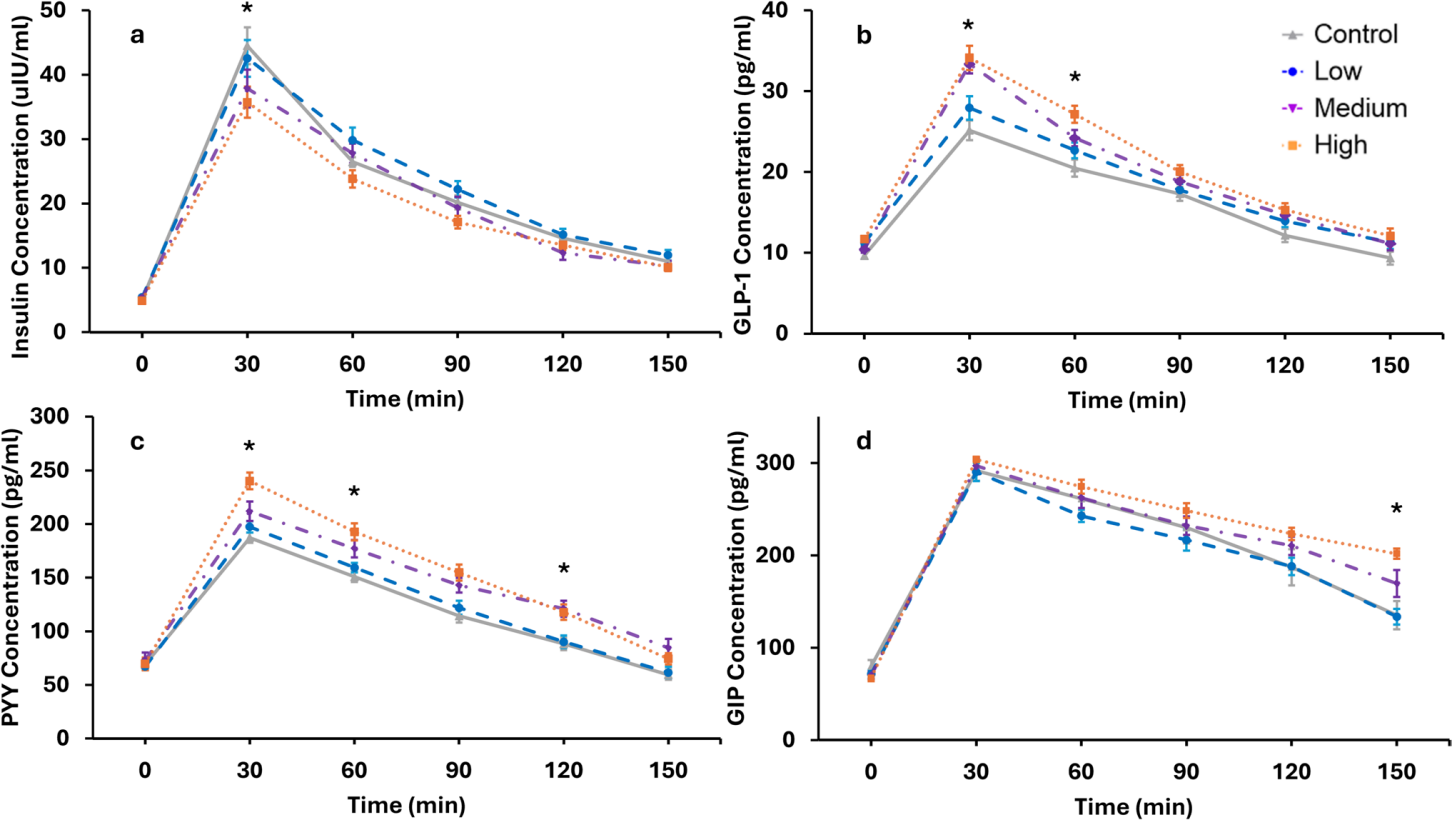
Postprandial **a**: insulin (n = 22), **b**: GLP-1 (n = 22), **c**: PYY (n = 21) and **d**: GIP (n = 8) concentrations following ingestion of three different doses of anthocyanin blueberry drinks and a control beverage without anthocyanins. Data are presented as mean ± SE and * denotes a significant condition x time interaction

Data for postprandial hormone concentrations are displayed in Fig. 4. For insulin, there was a statistically significant main effect of time F(5, 236) = 15.667, *p* < 0.001 and a statistically significant condition x time interaction F(9, 149) = 3.102, *p* = 0.002 with the HBB condition significantly lower at 30-min compared to the control. There was no main effect of condition F(3, 157) = 1.132, *p* = 0.338, no main effect of visit F(3, 157) = 0.349, *p* = 0.790 and no interaction of visit x condition F(15, 261) = 0.320, *p* = 0.993.

For plasma GLP-1 there was a significant main effect of time F(5, 290) = 10.853, *p* < 0.001, a significant main effect of condition F(3, 272) = 13.649, *p* < 0.01 and a significant condition x time interaction F(15, 290) = 1.071, *p* = 0.005. Pairwise comparisons identified that the MBB and HBB were both significantly higher than the control and LBB but did not differ from each other. In addition, GLP-1 peaked at 30-min postprandial for the HBB and MBB compared to the LBB and control and at 60 min between the HBB and all other drinks. There was no main effect of visit F(3, 230) = 0.542, *p* = 0.654 however a significant visit x condition effect was observed F(9, 233) = 2.404, *p* = 0.013 such that GLP-1 was significantly lower for the control drink on visit 4.

For plasma PYY, there was a significant main effect of condition F(3, 417) = 27.712, *p* < 0.001 and a significant main effect of time F(5, 287) = 34.139, *p* < 0.001. For condition, pairwise comparisons identified that the HBB and MBB were both statistically higher than the control drink (*p* < 0.001). There was also a significant condition x time interaction F(15, 224) = 5.033, *p* = 0.002, whereby the HBB and the MBB were significantly higher than the control at 30, 60 and 120-min postprandial. No significant main effects of visit were observed F(3,175) = 5.099, *p* = 0.685 or interaction of visit x condition F(9, 213) = 1.116, *p* = 0.352.

For GIP analysis, only a subset of 8 participants’ plasma samples was evaluated. There was a significant main effect of time F(5, 96) = 165, *p* < 0.001 and a significant main effect of condition F(3, 96) = 7.677, *p* < 0.001. For the effect of condition, pairwise comparisons revealed a significant difference between the HBB and control, the HBB and LBB and the MBB and LBB. A significant condition x time interaction was also observed F(7, 96) = 3.949, *p* < 0.001 such that GIP which was significantly higher at 150-min postprandial in the HBB and MBB conditions compared to the LBB and control. There was no significant effect of visit F(3, 96) = 0.891, *p* = 0.449 or interaction of visit x condition F(15, 96) = 0.777, *p* = 0.699.

### Subjective measures of appetite and mood

Data from the VAS ratings revealed a significant main effect of time for all outcomes except stress which remained unaffected across the study day (Table 2). A significant main effect of condition on fullness F(3, 502) = 2.972, *p* = 0.031 reflected that fullness was significantly lower for the HBB compared to the LBB over the test morning (mean difference -8.593, SE 2.838, *p* = 0.016) despite being equicaloric. However, there was no effect of condition on hunger ratings F(3, 502) = 0.333, *p* = 0.801. In addition, visit also showed a significant main effect for analysis of contentedness, alertness and concentration. Visit 3 was associated with significantly lower contentedness and concentration compared to all other visits in addition to lower alertness compared to visit 4 only. There were no significant condition x time interactions for any of the other VAS ratings (Supplementary Fig. 2).

**Table 2:** Outcomes from LMM for all VAS ratings

Rating	Condition	Time	Visit	Condition x time
Hunger	F(3, 502) = 0.333, *p = *0.801	**F(6, 502) = 7.772, *p *< 0.001**	F(3, 502) = 0.454, *p = *0.715	F(18, 502) = 0.374, *p = *0.992
Fullness	**F(3, 502) = 2.972, *p = *0.031**	**F(5, 502) = 51.586, *p* < 0.001**	F(3, 502) = 2.247, *p = *0.082	F(18, 502) = 0.319, *p = *0.997
Content	F(3, 502) = 1.605, *p = *0.187	**F(6, 502) = 19.548, *p* < 0.001**	**F(3, 502) = 11.085, *p* < 0.001**	F(18, 502) = 0.326, *p = *0.996
Sleepy	F(3, 502) = 1.210, *p = *0.305	**F95, 502) = 4.928, *p* < 0.001**	F(3, 502) = 2.926, *p = *0.213	F(18, 502) = 0.478, *p = *0.967
Alertness	F(3, 502) = 2.655, *p = *0.408	**F(6, 502) = 6.870, *p* < 0.001**	**F(3, 502) = 5.751, *p* < 0.001**	F(18, 502) = 0.321, *p = *0.997
Concentration	F(3, 502) = 4.413, *p = *	**F(6, 502) = 15.497, p < 0.001**	**F(3, 502) = 2.786, *p = *0.040**	F(18, 502) = 0.368, *p = *0.992
Stress	F(3, 502) = 13.394, *p = *0.091	F(6, 502) = 0.542, *p = *0.776	F(3, 502) = 2.785, *p = *0.440	F(18, 502) = 0.777, *p = *0.728

Bold indicates significant differences

### Effect of BB on cognitive outcomes

There were no effects of condition, session, visit or interactions on accuracy, reaction time or false alarms in the RVIP task. Similarly, there were no effects of condition, visit or interactions on accuracy or reaction time for the Corsi task. However, the effect of session just failed to reach significance F(1, 128) = 3.831, *p* = 0.052, with an increase in accuracy after completion of all visits, indicative of a potential practice effect. A significant effect of session was observed for VVLT immediate recall (F(1, 128) = 5.939, *p* = 0.016) and for VVLT delayed recall (F(1, 126) = 16.16, *p* < 0.01), whereby significantly fewer words were recalled post drink irrespective of condition. Proactive interference significantly decreased post drink regardless of condition F(1, 126) = 21.196, *p* < 0.01. For retroactive interference (RI) the condition x session interaction failed to reach significance F(3, 126) = 2.495, *p* = 0.063. Pairwise comparisons revealed that RI increased post drink for the control and LBB conditions, however RI was maintained after the MBB and HBB. Data for the cognitive outcomes can be seen in the Supplementary information (Supplementary tables 3, 4 and 5).

### Polyphenol concentration

Analysis of participants urine samples revealed a non-significant, dose-dependent increase in polyphenol concentration between the blueberry drinks (Supplementary Fig. 3).

## Discussion

This study is the first to examine the dose-dependent response of wild blueberry intake on postprandial glucose and satiety markers, concomitantly with cognitive performance. Findings demonstrate that a high dose of freeze-dried blueberries effectively ameliorates postprandial glycaemic response following a high-carbohydrate meal. These results are consistent with prior research outlining that interventions enriched with anthocyanin-rich berries can modulate postprandial glucose excursion, particularly within a 30–60 min window. Notably, in healthy adults, the consumption of a berry nectar (mixture of blackcurrants and lingonberries [35]) or berry puree (bilberries, blackcurrants, cranberries and strawberries [36]) alongside 35 g sucrose significantly reduced plasma glucose and insulin levels compared to the control group. Similarly, Schell et al. [37] documented lower glucose in type 2 diabetic patients after consuming cranberry following a high-fat breakfast, although insulin and insulin resistance remained unaffected.

Anthocyanins have been shown to modulate glucose metabolism through several mechanisms, one of which is their inhibitory action on key enzymes responsible for starch hydrolysis such as salivary/pancreatic α-amylase and intestinal α-glucosidases [38]. However, these effects appear to be anthocyanin specific. For instance, a comparative study using a stimulated in vitro digestion model revealed that both blackcurrants and greencurrants inhibited salivary α-amylase at concentrations of 0.66, 6.6 and 66 μg/mL. However, only blackcurrant inhibited α-glucosidase (highest inhibition observed at 66 μg/mL) which might be due to the higher levels of delphinidin, cyanidin aglycones and cyanidin-3-glucoside (C3G) and delphinidin-3-glucoside (D3G) in blackcurrant, compared to the presence of cyanidin-3-arabinose (C3A) detected in the greencurrants [39]. Since salivary α-amylase inhibition occurs immediately, while α-glucosidase inhibition typically transpires between 40 min to 2 h post-ingestion, the presence of both aglycones and glycosides in anthocyanin-rich foods may effectively suppress both enzymes involved in glucose breakdown, and as a consequence attenuate postprandial glucose excursion beyond the initial 60-min phase. These findings align with observations from digestion studies which show that anthocyanin glycosides are hydrolysed to their respective aglycones during digestion, with further degradation into flavium cations and other phenolics [40]. In the current wild blueberry intervention samples, D3G, C3G as well as C3A and D3A constituted main anthocyanins, underscoring the potential of anthocyanins (and their degradation products produced during digestion) to modulate glucose metabolism across the gastrointestinal tract.

### Postprandial hormones and satiety

The regulation of appetite and body weight involves a complex interplay of molecular pathways governing satiety signalling, energy homeostasis, and nutrient sensing. Central to this regulation are the gastrointestinal hormones, GLP-1, PYY and GIP which control satiety responses and food intake, and which were measured here after acute ingestion of three doses of blueberries. Our findings elucidate the role of blueberry anthocyanins in modulating the secretion of these key gastrointestinal hormones, thereby demonstrating potential influence on short term appetite regulation and in the longer-term body weight. Specifically, the medium (300 mg) and high (450 mg) blueberry drinks significantly stimulated higher GLP-1 and PYY secretion 30 min after intake. Interestingly, our study reported benefits on postprandial hormones which have not been consistently observed before. In a recent study [15], consumption of a high dose of blueberry (400 mg anthocyanins) did not significantly alter gastrointestinal hormones when compared to a matched placebo. In contrast, an earlier study reported near significant effects on GLP-1 after a mixed berry intervention (berry puree) [36]. These discrepancies in outcomes may reflect differences in study design and dietary intervention such as the size and composition of the carbohydrate meal [41], the type of sugar (glucose, sucrose, fructose, maltodextrin etc.), and presence of other nutrients such as protein [42] which are all known to affect glycaemic parameters and incretin hormones.

Under healthy physiological conditions, GIP levels peak shortly after meal consumption and then decline rapidly. However, in the current study we observed a sustained increase in GIP following medium and high blueberry drink which aligns with prior findings observed in an acute study using a blackcurrant beverage, where GIP levels also remained heightened even at 120 min post consumption [9]. It is important to note that our GIP analysis was based on a limited number of plasma samples (n = 8), raising the possibility of low power and necessitating caution in interpreting the significance of the observed effects. GIP may play a supplementary role, alongside GLP and insulin, in enhancing satiety. An interesting finding in this study is that despite blueberry interventions influencing incretin hormones, there was no effect on subjective measures of hunger. This may be due to non-homeostatic (i.e. hedonic) feeding behaviour traits, not regulated by satiety signalling via incretin hormones, but by reward, emotional and cognitive processes. The corticolimbic circuits in humans (including the striatum, amygdala, insula, nucleus accumbens and orbitofrontal cortex) are implicated in hedonic eating. During a 12-week trial, daily consumption of cranberry juice (containing 281 mg proanthocyanidins and 59 mg anthocyanins) significantly increased cerebral blood flow (CBF) to the orbitofrontal cortex and nucleus accumbens during cognitive testing [43]. It is plausible that habitual intake of anthocyanin-containing berries or other products could modulate brain activity in regions involved with the hedonic regulation of eating.

However, subjective fullness was significantly lower in the HBB group. There is a need for controlled clinical trials to test these hypotheses.

### Gut microbiome

Several of the biomarkers within this study show significant differences between the 30–60 min timepoint after ingestion of the MBB and HBB alongside the high carbohydrate meal. However, the AUC data over the time course of the study day (~ 180 min) did not demonstrate a significant overall effect. Repeated intake of blueberries is correlated with improvements in endothelial function [44, 45], insulin sensitivity [12], cognitive function [14] and other cardiometabolic health parameters [46]. It is suggested that repeated intake of anthocyanins may increase gut bacteria that enhance conversion to bioactive metabolites [28]. For example, long term blueberry supplementation has been shown to upregulate the abundance of bacteria, such as *Coriobacteriales* which facilitate the metabolism of dietary polyphenols [47]. The increase in abundance of *Coriobacteriales* was correlated with a reduction in large LDL particles in the postprandial state, though other circulating biomarkers remained unchanged.

In a controlled mouse study, supplementation with blueberry-derived polyphenols, specifically anthocyanidin- and proanthocyanidin-rich extracts, significantly modulated the gut microbiota composition and improved metabolic outcomes in diet-induced obese mice [48]. Notably, faecal microbiota transplantation experiments demonstrated that germ-free mice receiving microbiota from polyphenol-supplemented donors exhibited reduced adiposity and enhanced insulin sensitivity compared to controls, despite consuming the same obesogenic diet. These findings provide evidence that dietary polyphenols can reshape gut microbial communities in a manner sufficient to mediate improvements in host metabolic health. This highlights the causal role of the gut microbiome in linking polyphenol intake to beneficial metabolic phenotypes, supporting microbiota-targeted strategies for the prevention and management of metabolic disorders.

### Cognition and hyperglycaemia

Poorly controlled glucose can be detrimental to cognitive performance and is associated with an increased risk of cognitive decline [49]. Several possible mechanisms which link abnormalities in glucose and cognitive impairment include the expression, regulation, and the activity of glucose transporters which can be disrupted during hyperglycaemia. These changes negatively affect glucose uptake and metabolism in the brain, resulting in impairment of synaptic plasticity, neurogenesis, and ultimately cognitive function. Data from the current study showed no significant effect of blueberry dose on cognitive function outcome. These findings are contradictory to previous literature, elucidating benefits to cognition in children, [50] middle-aged adults, [51, 52] and in older adults [53].

The lack of statistically significant acute effects of blueberry at these doses on cognitive outcomes and treatment-related interactions in insulin or glucose suggests that there may be different mechanisms by which blueberry anthocyanins modulate cognitive function in healthy individuals. It is plausible that any cognitive benefits observed in earlier studies could be attributed to improvements in CBF. Improvements in working memory following anthocyanin ingestion, concurrent with increases in CBF have previously been observed in healthy older adults [54]. Measuring CBF was outside the scope of the current study, and blood pressure was measured to assess vascular function.

The participants in this study were relatively young and healthy which may have limited capacity for short-term cognitive enhancement, consistent with other studies of similar populations [28]. Furthermore, mechanisms underlying cognitive benefits such as upregulation of brain-derived neurotrophic factor, improved neurovascular coupling or gut-derived metabolite effects likely require repeated exposure to blueberry anthocyanins to exert strong effects.

Acutely, no differences in blood pressure were discerned between the beverages, consistent with previous observations [13]. Nethertheless, it is important to acknowledge that the lack of an effect on blood pressure in our study does not negate the accumulating evidence which suggests that anthocyanins may confer beneficial effects on blood pressure regulation in individuals with hypertension or elevated blood pressure [55] or may prevent the development of high blood pressure [56].

## Limitations

It is plausible that the dietary intervention did not contain enough calories to elicit differences in subjective appetite assessment. When assessing appetite, it is recommended that the pre-load meal should be substantial and mirror typical dietary habits [57]. In the current investigation, the pre-load meal amounted to 280 kcal (+ the test beverage) which was higher than previous similar studies [38]. Furthermore, the initial post-consumption appetite evaluation occurred at + 30 min to permit measures of appetite hormones and assessment of cognitive function. Recommendations are to assess subjective appetite immediately post-consumption of the test item, as well as at regular intervals of 15 min (up to 1 h). However, in the interests of minimising participant burden, the decision was made to reduce the number of subjective ratings completed by participants. Subsequently, it is conceivable that the energy density of the test meal, coupled with the timing of the initial appetite assessment, might have led to a modest reduction in hunger and desire to eat, that were not captured by the experimental protocol. In addition, low representation of males (n = 2) who tend to show greater sensitivity to hunger compared to females (n = 22) may further limit the generalizability of our findings.

Finally, we did not monitor habitual anthocyanin intake and only requested a 24-h anthocyanin restriction period before each visit. As anthocyanin metabolites can persist in circulation for several days [58], residual intake may have added variability to baseline levels and masked intervention effects.

## Conclusion

Our findings highlight the potential of anthocyanin-rich blueberries to modulate postprandial glycaemic response and gastrointestinal hormone secretion. Specifically, higher doses of freeze-dried blueberries were effective in reducing postprandial glucose levels and enhancing GLP-1 and PYY secretion, suggesting a beneficial role in the modulation of glucose metabolism, with potential impact beyond the acute effects observed. These findings align with and extend previous research on the physiological benefits of anthocyanin-rich berries but also highlight the important influence of study design and population, and intervention type on target outcomes.

The lack of effects observed on subjective measures of appetite and cognitive performance in the current study, underscores the complexity of non-homeostatic influences and potential mechanistic differences to modulate cognitive performance. Future anthocyanin intervention studies are merited to explore satiety hormones, subjective appetite and cognitive performance in vulnerable samples such as those with less well controlled glycaemia.

## Supplementary Information

Below is the link to the electronic supplementary material.

Supplementary file1 (DOCX 370 KB)

## Data Availability

Anonymised data are available from the corresponding author upon reasonable request.
