# Supplementary material for: A randomised, double-blind, placebo-controlled trial to assess the postprandial dose-dependent effects of wild blueberries on metabolic and cognitive outcomes following a high-carbohydrate breakfast
Source: Eur J Nutr. 2026 May 26;65(4):138. doi: 10.1007/s00394-026-03974-0 (PMC13212708; doi:10.1007/s00394-026-03974-0)
Supplement: Supplementary file 1 — Supplementary file1 (DOCX 370 KB) [file 394_2026_3974_MOESM1_ESM.docx]

**A randomised, double-blind, placebo-controlled trial to assess dose-dependent effects of wild blueberries on glucose, satiety, blood pressure and cognition**

**Lucy R Ellis^a,b,c^, Dominic O’Connor^d^, Haseena Khan^b^, Louise Dye^a,b,e^, Christine Boesch^b^***

^a^ School of Psychology, University of Leeds, Leeds, UK;

^b^ School of Food Science and Nutrition, University of Leeds, Leeds, UK;

^c^Carnegie School of Sport, Leeds Beckett University, Leeds, UK;

^d^ Division of Psychology and Mental Health, University of Manchester, Manchester, UK;

^e^ Institute of Sustainable Food, University of Sheffield, Sheffield, UK

* Corresponding author; c.bosch@leeds.ac.uk

**Supplementary Material**

**Supplementary Table 1.** Nutritional composition of the standardised meals

|  | **Standardised evening meal (a)** | **Standardised breakfast (b)** |
| --- | --- | --- |
| Energy (kcal) | 788 | 280 |
| Total carbohydrate (g) | 90 | 53.4 |
| Sugar (g) | 4.2 | 3.4 |
| Fibre (g) | 7 | 2.6 |
| Protein (g) | 36 | 10.2 |
| Fat (g) | 30 | 2.4 |

a – Standardised evening meal consisted of a 320g margherita pizza (Goodfellas), 1x 320ml can of diet coke and 1, sugar free Roundtree jelly pot. b – Standardised breakfast served with each test drink, consisting of two slices of Warburtons super toastie, extra thick white bread (toasted) and 15g margarine (Flora buttery spread). Macronutrient content of each meal was taken from the back of pack label.

**Supplementary Table 2.** Macronutrient and anthocyanin content of the test beverages

|  | **Control** | **Low dose (LBB)** | **Medium Dose (MBB)** | **High Dose**  **(HBB)** |
| --- | --- | --- | --- | --- |
| Freeze dried blueberry powder (g) | 0 | 10 | 20 | 30 |
| Energy (kcal) | 109.2 | 110.05 | 110.5 | 110.68 |
| Total carbohydrate (g) | 27.3 | 27.2 | 27.1 | 27 |
| Sugar (g) | 19.5 | 19.7 | 19.9 | 20.1 |
| Fibre (g) | 4.8 | 4.7 | 4.6 | 4.5 |
| Fat (g) | 0 | 0.05 | 0.1 | 0.12 |
| Protein (g) | 0 | 0.2 | 0.3 | 0.4 |
| Anthocyanins (mg) * | 0 | 145.5 | 291 | 436.5 |

Analysis of macronutrient contents are from the certificate of analysis provided by Merieux NutriSciences, Silliker Inc, Crete, IL. * Values calculated from certificate of analysis provided by Plants for Human Health Institute, North Carolina State University, North Carolina Research Campus, Kannapolis, NC (conducted using HPLC)

**Supplementary Table 3.** Results of the Rapid Visual Information Processing Task

| **Measure** | **Time** | **Control** | **150 mg** | **300 mg** | **450 mg** |
| --- | --- | --- | --- | --- | --- |
| Reaction Time (ms) | Baseline | 382.32 ± 12.02 | 390.50 ± 7.68 | 393.55 ± 8.33 | 393.43 ± 7.67 |
|  | Post | 380.12 ± 12.94 | 389.77 ± 8.79 | 394.47 ± 7.56 | 387.75 ± 9.24 |
| Number Correct (%) | Baseline | 35.95 ± 2.59 | 34.57 ± 3.06 | 37.25 ± 3.66 | 31.16 ± 3.46 |
|  | Post | 31.81 ± 3.77 | 38.12 ± 3.72 | 38.84 ± 3.79 | 38.84 ± 4.00 |
| False Alarms | Baseline | 12.64 ± 2.64 | 12.64 ± 2.84 | 12.05 ± 2.80 | 14.00 ± 2.08 |
|  | Post | 13.09 ± 2.52 | 14.55 ± 3.94 | 12.68 ± 3.60 | 12.55 ± 2.69 |

Data from the RVIP presented as means ± SE.

**Supplementary Table 4.** Results of the Corsi Block Tapping Task

| **Measure** | **Time** | **Control** | **150 mg** | **300 mg** | **450 mg** |
| --- | --- | --- | --- | --- | --- |
| Reaction Time (ms) | Baseline | 706.66 ± 44.70 | 680.90 ± 55.93 | 642.23 ± 55.56 | 628.19 ± 33.27 |
|  | Post | 655.86 ± 48.82 | 638.80 ± 47.48 | 570.49 ± 45.16 | 621.86 ± 44.64 |
| Accuracy (%) | Baseline | 76.44 ± 2.50 | 78.22 ± 3.36 | 76.19 ± 3.73 | 74.55 ± 4.58 |
|  | Post | 78.42 ± 3.29 | 78.87 ± 3.82 | 79.66 ± 2.96 | 73.21 ± 5.31 |

Data from the Corsi task presented as means ± SE.

**Supplementary Table 5.** Results of the Visual Verbal Learning Task

| **Measure** | **Time** | **Control** | **150 mg** | **300 mg** | **450 mg** |
| --- | --- | --- | --- | --- | --- |
| Total Words Recalled (A1-A3) | Baseline | 32.67 ± 0.96 | 32.46 ± 0.90 | 31.92 ± 1.05 | 30.83 ± 1.33 |
|  | Post | 29.17 ± 1.39 | 30.08 ± 1.28 | 31.00 ± 1.10 | 30.67 ± 1.16 |
| Delayed Words | Baseline | 10.29 ± 0.58 | 10.58 ± 0.58 | 10.42 ± 0.58 | 10.71 ± 0.58 |
|  | Post | 8.92 ± 0.78 | 9.54 ± 0.78 | 9.04 ± 0.78 | 9.83 ± 0.78 |
| Proactive Interference | Baseline | 1.88 ± 0.52 | 1.38 ± 0.68 | 1.71 ± 0.48 | 1.29 ± 0.46 |
|  | Post | 0.04 ± 0.37 | 0.42 ± 0.55 | 0.17 ± 0.45 | −0.50 ± 0.63 |
| Retroactive Interference | Baseline | 2.38 ± 0.76 | 2.13 ± 0.54 | 2.29 ± 0.46 | 2.50 ± 0.43 |
|  | Post | 2.92 ± 0.49 | 2.63 ± 0.45 | 2.38 ± 0.38 | 2.42 ± 0.58 |

Data from the VVLT presented as means ± SE.


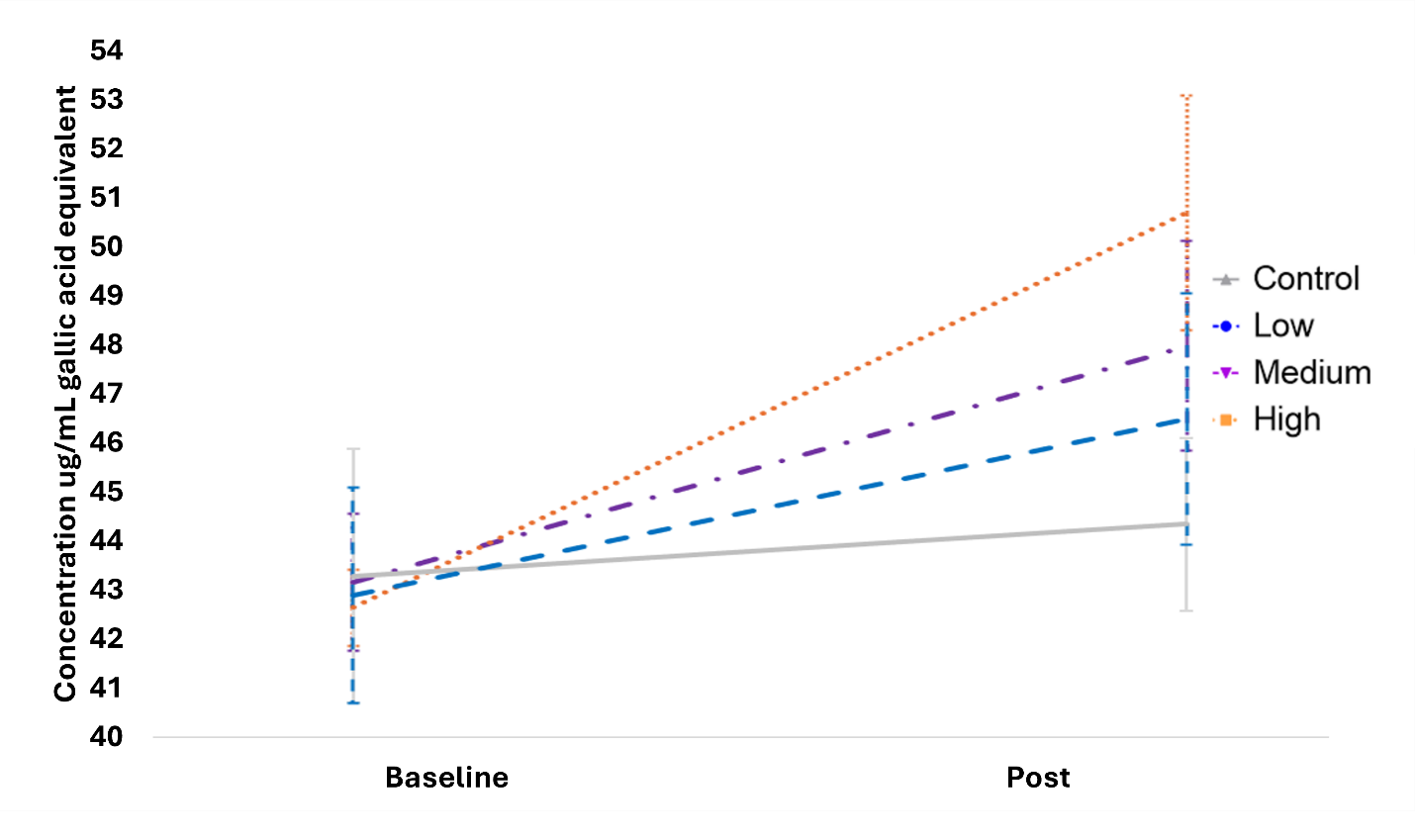


**Supplementary Figure 1.** Total polyphenol concentration of each test drink as analysed by fast blue analysis. Data is presented as means ± SE.


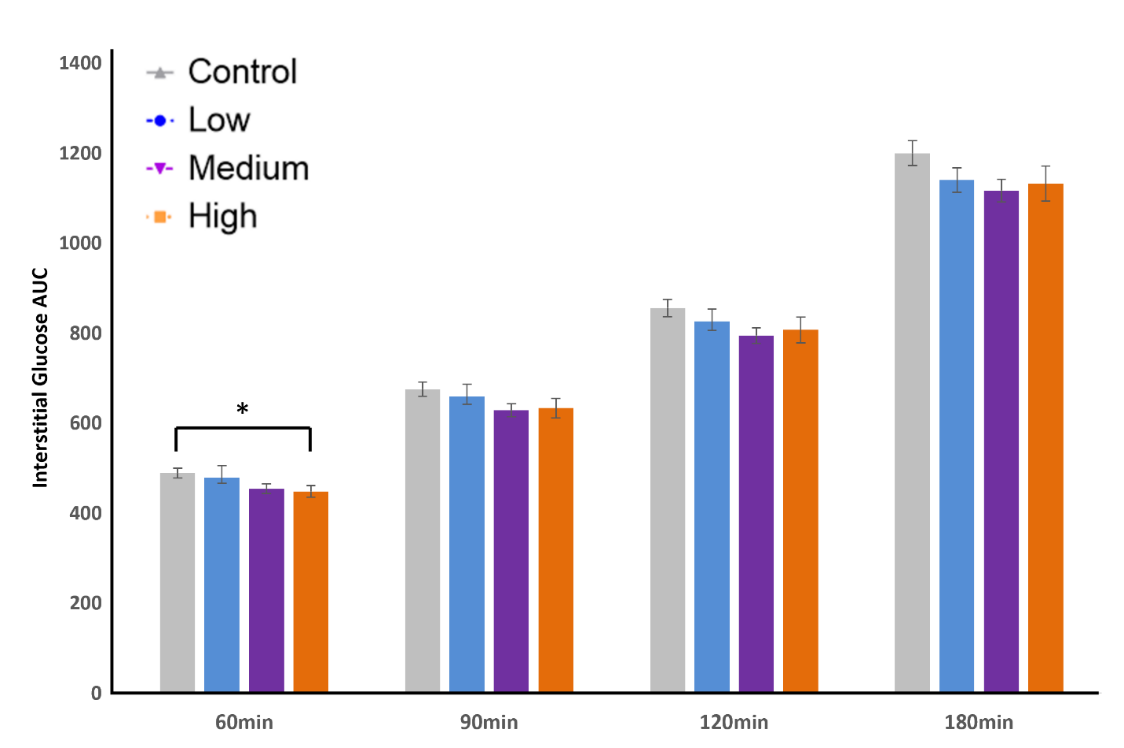


**Supplementary Figure 2. AUC values calculated at different time points using trapezoidal method. A significant difference between the control and HBB was observed at 30 min postprandial. No other significant effects were observed. Data is presented as means** ± SE.


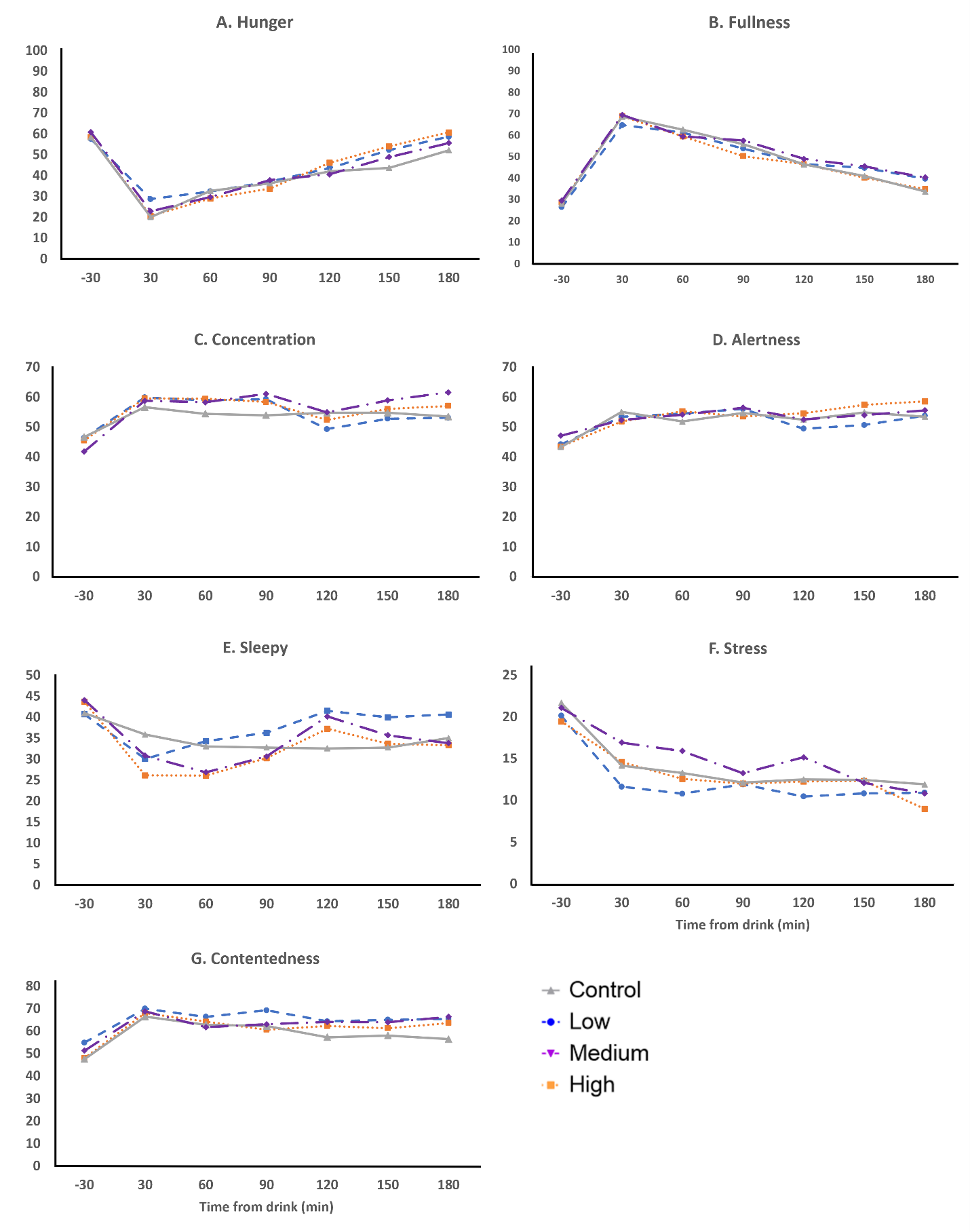


**Supplementary Figure 3.** Outcomes from each individual VAS. Data is presented as means.
